# The impact of depression and anxiety on the correlation between somatic symptom disorder and subjective cognitive decline in the Chinese elderly population: an exploration by simple, serial, and moderated mediation models

**DOI:** 10.3389/fpsyg.2025.1545325

**Published:** 2025-02-14

**Authors:** Yongsen Lin, Pingzhen Lin, Guiying Zeng, Shufang Wu, Ronghua Chen, Yingchun Xiao, Hongbin Chen

**Affiliations:** ^1^Department of Neurology, Quanzhou First Hospital Affiliated to Fujian Medical University, Quanzhou, China; ^2^Nursing Department, Quanzhou First Hospital Affiliated to Fujian Medical University, Quanzhou, China; ^3^Department of Neurology, Fujian Medical University Union Hospital, Fuzhou, China

**Keywords:** somatic symptom disorder, subjective cognitive decline, depression, anxiety, mediation

## Abstract

**Background:**

Somatic symptom disorder (SSD) exacerbates subjective cognitive decline (SCD). This association can be significantly impacted by the mental well-being of the SSD patients. However, the underlying mechanisms remain obscure.

**Methods:**

A total of 525 elderly patients, who visited the Department of Neurology of Fujian Medical University Union Hospital between October 2017 and August 2024, and the Department of Neurology of Quanzhou First Hospital Affiliated to Fujian Medical University between August 2022 and August 2024, were included in the study. Data of sociodemographics, medical history, somatic symptom disorder, subjective cognitive decline, depression, and anxiety were analyzed by simple, serial and moderated mediation models to evaluate the impact of depression and anxiety on the linkage between SSD and SCD.

**Results:**

SSD significantly affected SCD. Simple mediation analysis showed that depression and anxiety significantly mediated the association between SSD and SCD (β_depression_ = 0.079, 95% *CI*: 0.030 to 0.132; β_anxiety_ = 0.058, 95% *CI*: 0.031 to 0.093). Serial mediation analyses indicated that the worsening of SSD exacerbates anxiety, in turn aggravating depressive symptoms and SCD (β*_b_* = 0.044, 95% *CI*: 0.026 to 0.069). Moderated mediation model revealed that the depressive symptoms-conferred mediation of the correlation between SSD and SCD was moderated by anxiety symptoms (β = −0.073, 95% *CI*: −0.131 to −0.014).

**Conclusion:**

These findings provided new insights into possible avenues for prevention and intervention of SCD through SSD-based treatments with a multifaceted approach to psychiatric disorders.

## 1 Introduction

Subjective cognitive decline (SCD) refers to cognitive decline without the presence of objective impairment in cognitive evaluation (Jessen et al., 2020). The prevalence of a certain degree of cognitive impairment has been documented in about 50 to 80% of older individuals who score within the normal range in cognitive assessments (van Harten et al., 2018). Declines in several cognitive domains, such as attention, executive function and visuospatial skills, can occur with the advancing age (Hoogendam et al., 2014). Other studies have also evidenced that cognitively normal people who present SCD complaints are at a higher risk of developing subsequent objective cognitive decline, leading to mild cognitive impairment (MCI) and dementia (Mitchell et al., 2014; Slot et al., 2019). Still others have argued that SCD is indicative of the primary symptoms of preclinical Alzheimer’s disease (AD), serving to aid the early diagnosis of AD (Ossenkoppele and Jagust, 2017) and that SCD evaluation can facilitate early intervention for those who are at a risk of developing dementia in their late life (Olivari et al., 2021). Due to the significant burden that cognitive decline can place on society, it is of great significance to investigate the risk factors that impact the development of SCD.

Currently, a body of studies demonstrate that the onset of SCD is highly correlated with various psychiatric disorders, particularly depression and anxiety (Jenkins et al., 2021; Jessen et al., 2020). Some studies suggest that somatic disorders, pain, and certain personality traits, such as neuroticism, may contribute to or exacerbate age-related cognitive changes (Comijs et al., 2022; Horgas et al., 2022). Still some report that individuals younger than 60 years are more likely to experience SCD and indicate that the condition is related to some potentially reversible factors such as depression (Jessen et al., 2020). Taken together, these findings illuminate the possibility of SCD prevention by addressing the psychological well-being and somatic symptoms of the potential patients.

Somatic symptom disorder (SSD) is characterized by a persistent physical symptom and related psychological impairment at affective, cognitive, or behavioral levels, including physical symptoms leading to dysfunction in daily life and health-related anxiety (American Psychiatric Association, 2013). A recent review indicates that the prevalence of SSD among outpatients in Chinese hospitals is approximately 33.8% (Cao et al., 2020). The SSD prevalence is associated with a reduction in the quality of life of the affected individuals, a considerable increase in healthcare utilization, and a high frequency of medical visits for an array of symptoms, including pain, fatigue, and gastrointestinal discomfort, which has led to an elevated global disease burden (Henningsen, 2018). Moreover, evidence suggests that individuals diagnosed with SSD are at an elevated risk for suicidal attempts (Torres et al., 2021). Accordingly, an early diagnosis of and intervention for SSD should prove most beneficial for the afflicted individuals.

Despite the growing incidence of SSD and the high cost on the families and society, the current treatment for SSD is far from satisfactory and SSD-related factors are still needed to be pinpointed to facilitate timely interventions (Jongsma et al., 2023). Studies have shown that the incidence and severity of SSD are higher in the elderly than in the non-elderly (Wu et al., 2022) and that SSD is often comorbid with anxiety, depression, and sleep disorders, with depression being the most common (Ionescu et al., 2021; Kohlmann et al., 2016; Wu et al., 2023). Compared with youth depression, late-life depression has higher rates of disability and residual symptoms, and the severity of depressive symptoms is closely related to white matter lesions (Taylor et al., 2013). Recent studies have shown that SSD can significantly affect cognitive function in the elderly population, mainly manifested as decline in cognitive functions, such as attention, working memory and executive ability (Huang et al., 2021; Kim et al., 2021; Lee et al., 2010). Collectively, these findings suggest that these somatic factors may be involved in the association of SSD and cognitive decline and that a further exploration of these factors may pave the way for timely intervention.

To date, most studies of the effects of somatic symptoms on cognitive decline have focused on objective cognitive dysfunction. They have largely failed to probe into SCD and the underlying involvement of psychiatric disorders. For example, a previous study investigated the effects of anxiety and depression on neurocognitive functioning in patients with SSD (de Vroege et al., 2017) with a neuropsychological battery of tests that only assess objective cognitive abilities, including basic cognitive processes, executive functioning, and memory processes. Another recent study investigated the relationship between SSD and subjective cognitive functioning but only assessed patients’ executive functioning and failed to consider the role of psychiatric disorders at play (Huang et al., 2021). So far, no studies have investigated the direct or indirect effects of SSD on SCD and considered the potential mediating or moderating role of depression and anxiety. Therefore, the current study attempted to examine the relationship between SSD and SCD and to explore the role of depression and anxiety in their association. The following hypotheses were therefore formulated (Giannouli et al., 2022; Skoog, 2011; Xu et al., 2021):

*Hypothesis 1*: Depression or anxiety mediates the relationship between SSD and SCD, respectively.

*Hypothesis 2*: Depression and anxiety together play a serial mediating role in the relationship between SSD and SCD.

*Hypothesis 3*: Depression or anxiety may, respectively, moderate the anxiety or depression-mediated SSD-SCD association.

## 2 Materials and methods

### 2.1 Research participants

This study recruited a total of 525 elderly patients who visited the Department of Neurology at Fujian Medical University Union Hospital between October 2017 and August 2024, and the Department of Neurology at Quanzhou First Hospital Affiliated to Fujian Medical University between August 2022 and August 2024. The patients were excluded from the study due to any of the following exclusion criteria: (1) serious heart, liver, kidney, or respiratory diseases; (2) Alzheimer’s disease, Parkinson’s disease, intracranial tumors, or other neurological conditions; (3) inability to complete cranial MRI examination. The study protocol was approved by the Ethics Committee at Fujian Medical University Union Hospital (Approval No.: 2013038) and Quanzhou First Hospital Affiliated to Fujian Medical University (Approval No.: 2021160). Informed consent was obtained from all patients enrolled.

Participants underwent a series of scale assessments by neurologists trained in scale assessment and data collected included socio-demographic characteristics (such as age, sex, disease duration and education level), Fazekas grade (Fazekas et al., 1987), chronic disease, mental health (depression and anxiety), SSD score and SCD score. As this was cross-sectional and no further follow-up of study subjects was performed, there was no dropout involved and since we used a face-to-face questionnaire administered by physicians, the questionnaire response rate was 100%.

### 2.2 Assessments

#### 2.2.1 Somatic symptom disorder

Somatic symptoms disorder was examined using the patient health questionnaire-15 (PHQ-15), which is commonly used to assess somatic symptoms (Kroenke et al., 2002). The scale includes three primary factors, including cardiopulmonary, pain and gastrointestinal factors, each factor consisting of 5 items and each item with a scoring range from 0 to 5. The total score was 75, with a higher total score indicating a severer somatic symptom disturbance (Liao et al., 2016). A PHQ-15 score of 5 or higher indicates somatic symptoms (AUC = 0.725). In addition, PHQ-15 scores of 5, 10 and 15 correspond, respectively, to cut-off points for mild, moderate and severe somatic symptoms (Liao et al., 2016).

#### 2.2.2 Depression

Depression severity in patients was assessed using the 17-item Hamilton Rating Scale for Depression (HAMD-17), each item with a scoring range from 0 to 4, in which a higher score indicates a greater severity. The HAMD-17 has good reliability and stability and can be used to screen depressive symptoms in primary care populations (Zimmerman et al., 2013). The HAMD-17 measures depressive symptoms on a scale from no depression (0–7) to mild depression (8–16), moderate depression (17–23), and major depression (≥24) (Zimmerman et al., 2013).

#### 2.2.3 Anxiety

Anxiety symptoms in enrolled patients were assessed using the 14-item Hamilton Anxiety Rating Scale (HAMA-14), which has good reliability and stability and is widely used to measure anxiety in patients with depressive symptoms (Maier et al., 1988). The HAMA-14 scale consists of 14 items assessing nervousness, insomnia, somatic and gastrointestinal symptoms, each item with a scoring range from 0 to 4, in which a higher score indicates a greater severity. A total score of less than 7 indicates no anxiety; 14 or more suggests definitely anxious; 21 or more markedly anxious; and 29 or more severely anxious (Maier et al., 1988).

#### 2.2.4 Subjective cognitive decline

Perceived Deficits Questionnaire for Depression (PDQ-D) evaluates four cognitive domains: attention/concentration, retrospective memory, prospective memory, and planning/organization. It can effectively assess subjective cognitive dysfunction in patients with major depression, making it psychometrically valuable (Shi et al., 2017). The 5-item Perceived Deficits Questionnaire for Depression (PDQ-5-D), a simplified version of the PDQ-D, which has 5 items, each item with a scoring range of 0–4, amounting to a total score of 20. This questionnaire is effective in assessing subjective cognitive function in depressed patients, with a high score indicating severer subjective cognitive deficits (Fehnel et al., 2013).

#### 2.2.5 Demographic characteristics and medical history

Sociodemographic characteristics such as sex (male/female), age (in years) and education background were collected. Individuals were categorized into two age groups: under 70 and over 70. The education level was categorized as primary school or less (≤6 years), junior high school (6–9 years) and senior high school or more (≥9 years). Medical history included Fazekas grade, duration of illness, and concurrent chronic illness. Duration of illness referred to the duration of the somatic symptom disorder state and was divided into a state of less than 3 years and that of more than 3 years. Chronic conditions associated with the illness included hypertension, diabetes and cardiovascular disorders, and patients were categorized primarily according to their previous history of these chronic conditions when receiving the questionnaire.

#### 2.2.6 Statistical analysis

The study employed t-tests and one-way ANOVA to examine SCD disparities in demographic characteristics and pertinent medical history (Xu et al., 2021). Spearman correlation analysis was conducted to test associations among essential variables. In determining the sample size, the study set the power at 0.9 and the type I error rate at 0.05 (two-tailed). A correlation coefficient (r) of 0.16 was chosen as the effect size in this study since the correlation coefficients for any two variables between HAMD, HAMA, PHQ-15, and PDQ-5-D were above 0.16, with a larger effect size indicating a smaller sample size. As a result, to achieve an effect size of 0.16, the theoretically estimated sample size was 331, which corresponded to a “small effect size.” A total of 525 participants were ultimately enrolled in the study, satisfying the necessary sample size. The “pwr” analysis package within the R language pwr.r.test section was adopted for the sample size analysis (Xu et al., 2021).

The macro-program PROCESS 3.5 developed by Hayes (2013) was used to explore simple, serial, and moderated mediation models, which were used to test our hypotheses and explore the pathways that link SSD to SCD (Hayes, 2013; Xu et al., 2021). Of the three candidate models (Model 4, 5, and 6) for the mediation analysis in the macro-program PROCESS, Model 4 (simple mediation) was adopted to explore the mediating role of depression and anxiety in the linkage between SSD and SCD (Preacher and Hayes, 2008; Xu et al., 2021). Subsequently, Model 6 (serial mediation) was chosen to investigate the relationship between mediators, which systematically examined the mediators in sequence and assessed the indirect effects of each mediator independently (Xu et al., 2021). Finally, for the 2 moderated mediation models (Model 14 and 15) in the macro-program PROCESS regarding the b-path-moderated mediation, Model 14 (moderated mediation via the b-path rather than the c’-path/direct effect) was employed to analyze the moderating role of depression in mediating the SSD-anxiety-SCD linkage and that of anxiety in mediating the SSD-depression-SCD correlation (Hayes, 2013).

If statistical significance was confirmed for SCD by one-way ANOVA test in the analysis of demographic characteristics and relevant medical history, further screening was performed by multiple linear regression method. The statistically significant variables were then included as covariates in the model. Follow-up bootstrap analyses were conducted to explore all indirect effects using 5,000 bootstrap samples (Preacher and Hayes, 2008) and 95% bias-corrected confidence intervals, in which the statistical significance was indicated if zero was not included (Mallinckrodt et al., 2006). Non-standardized coefficients (β) and 95% confidence intervals with their standard errors (SE) were reported for each model. All statistical analyses were conducted using IBM SPSS 25.0 software (SPSS Inc., IL, United States) and all tests were two-sided at a significance level of 0.05 (two-tailed) (Xu et al., 2021).

## 3 Results

### 3.1 Preliminary analyses

The demographic characteristics, medical history, and distribution of SCD among the 525 participants are summarized in Table 1. The scores for SCD were significantly associated with age, educational level, disease duration, Fazekas grade and somatic symptom severity, with a mean SCD score of 10.92. After the univariate analysis, the crucial factors were further screened by multiple linear regression. The results are outlined in Supplementary Table S1. Multivariate analyses reported a statistical significance for disease duration and Fazekas grade, which were therefore included as covariates in the model. The results of multicollinearity showed that the VIF for age, education, disease duration, Fazekas grade, and somatic symptom severity was all less than 5, indicating that no multicollinearity between these factors and SCD.

**Table 1:** Demographic characteristics and medical history of the included participants with subjective cognitive decline (*N* = 525).

Variables	*M* ± SD	*N* (%)^a^	SCD (M ± SD)	*t/F* ^b^	*p*
Gender	N/A			−1.344	0.180
Male		260 (49.52%)	10.70 ± 3.55		
Female		265 (50.48%)	11.13 ± 3.87		
Age (year)	71.01 ± 5.83			−7.349	<0.001^***^
≤70		238 (45.33%)	9.67 ± 3.42		
>70		287 (54.67%)	11.95 ± 3.64		
Education level (year)	7.65 ± 2.40			5.094	0.006^**^
≤6		189 (36%)	11.44 ± 3.90		
More than 6 less than or equal to 9		248 (47.24%)	10.86 ± 3.48		
>9		88 (16.76%)	9.93 ± 3.81		
Disease duration (year)	4.27 ± 2.61			27.762	<0.001^***^
≤3		233 (44.38%)	9.70 ± 3.32		
More than 3 less than or equal to 6		184 (35.05%)	11.49 ± 3.71		
>6		108 (20.57%)	12.56 ± 3.70		
Fazekas scores	N/A			27.403	<0.001^***^
1		149 (28.38)	9.40 ± 3.49		
2		213 (40.57%)	10.86 ± 3.45		
3		163 (31.05%)	12.37 ± 3.72		
Hypertension	N/A			−1.858	0.064
Yes		232 (44.19%)	11.25 ± 3.97		
No		293 (55.81%)	10.65 ± 3.49		
Diabetes	N/A			−1.437	0.151
Yes		171 (32.57%)	11.25 ± 4.02		
No		354 (67.43%)	10.75 ± 3.55		
Cardiovascular disorders	N/A			−0.580	0.562
Yes		204 (38.86%)	11.03 ± 3.45		
No		321 (61.14%)	10.84 ± 3.88		
Somatic symptom severity	11.00 ± 3.77			39.886	<0.001^***^
None to mild (0–9)		199 (37.90%)	9.35 ± 3.20		
Moderate (10–14)		224 (42.67%)	11.38 ± 3.65		
Severe (≥15)		102 (19.43%)	12.96 ± 3.57		
Depression severity	14.93 ± 4.63			48.353	<0.001^***^
None to mild (0–16)		310 (59.05%)	9.75 ± 3.47		
Moderate (17–23)		206 (39.24%)	12.47 ± 3.31		
Severe (≥24)		9 (1.71%)	15.78 ± 4.35		
Anxiety severity	15.82 ± 4.81			22.990	<0.001^***^
None to mild (0–14)		207 (39.43%)	9.77 ± 3.53		
Moderate (15–21)		253 (48.19%)	11.32 ± 3.62		
Severe (≥22)		65 (12.38%)	12.97 ± 3.48		

a Numbers are unweighted, but percentages are weighted.

b *t, t test; F, one way ANOVA.*

* Significance at the 0.05 level (two-tailed); ** Significance at the 0.01 level (two-tailed); *** Significance at the 0.001 level (two-tailed).

*M ± SD, Values are presented as mean ± standard deviation.*

The correlations among SCD, anxiety, depressive symptoms and SSD are presented in Supplementary Table S2. SSD was positively correlated with anxiety symptoms (*r* = 0.316, *p* < 0.001), depressive symptoms (*r* = 0.169, *p* < 0.001), and SCD (*r* = 0.374, *p* < 0.001). Anxiety symptoms was positively correlated with depressive symptoms (*r* = 0.339, *p* < 0.001), and SCD (*r* = 0.287, *p* < 0.001). Depressive symptoms were positively correlated with SCD (*r* = 0.523, *p* < 0.001).

### 3.2 Simple mediation model

**Figure 1:**
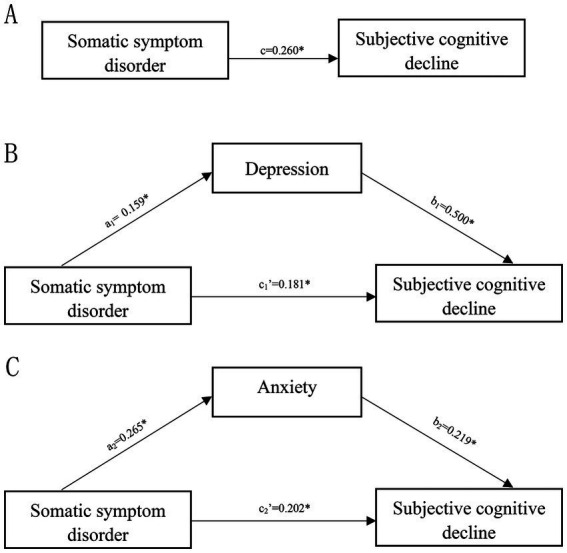
Simple mediation model. **(A)** The total effect of somatic symptom disorder on subjective cognitive decline; **(B)** the direct and indirect effects of depression on the association of somatic symptom disorder with subjective cognitive decline. **(C)** The direct and indirect effects of anxiety on the association of somatic symptom disorder with subjective cognitive decline. Numbers associated with a, b, c, and c′ are unstandardized regression coefficients. *Significance at the 0.05 level (two-tailed).

The statistical analysis revealed a significant impact of SSD on SCD (*c* = 0.260, 95% *CI*: 0.171 to 0.349) (Figure 1A). The mediating role of depression and anxiety was analyzed separately. The results indicated significant mediation effects of depression (a1 × b1 = 0.079, 95% *CI*: 0.030 to 0.132) and anxiety (a2 × b2 = 0.058, 95% *CI*: 0.031 to 0.093) (Figures 1B,C). The details of the effect value of the simple mediation model are presented in Supplementary Table S3. The results showed that SSD exerted not only direct effects on SCD but also significant indirect effects on SCD through depression and anxiety symptoms, with depression being a stronger mediator than anxiety.

### 3.3 Serial mediation model

**Figure 2:**
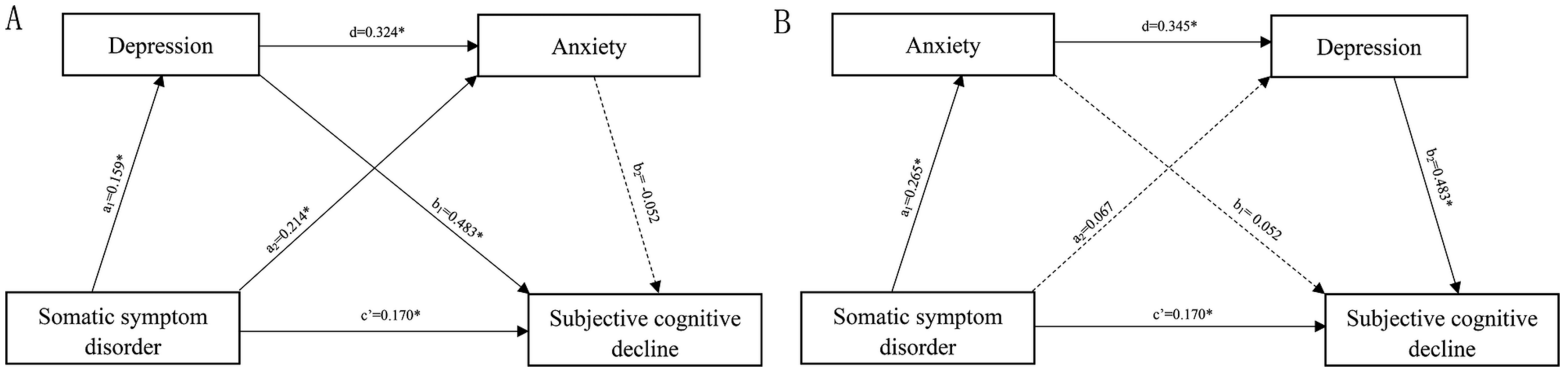
Serial mediation model. **(A)** The pathway of somatic symptom disorder →depression→ anxiety → subjective cognitive decline and **(B)** the pathway of somatic symptom disorder → anxiety→ depression → subjective cognitive decline. Numbers associated with a, b, c’, and d are unstandardized regression coefficients. *Significance at the 0.05 level (two-tailed).

The coefficients and significance of each path are presented in Figure 2 and the bootstrap results for the indirect effect are detailed in Table 2. The indirect effect path (SSD → depression→ anxiety→ SCD) (β*_a_* = 0.003, 95% *CI*: −0.001 to 0.009) of the serial mediation model was no significant, while the alternative indirect effect path (SSD → anxiety → depression → SCD) (β*_b_* = 0.044, 95% *CI*: 0.026 to 0.069) of the serial mediation model was significant. This suggests that the worsening of SSD exacerbates anxiety, in turn aggravating depressive symptoms and SCD. However, SSD produced no direct effect on depression, which may be attributed to the effect of anxiety on depression.

**Table 2:** Comparisons of the bootstrap results of the serial mediation models a and b.

Path	Estimate	SE	*t*	*p*	Bootstrap LLCI^c^	Bootstrap ULCI^c^
Total effect	0.260	0.045	5.742	<0.001^***^	0.171	0.349
Direct effect	0.170	0.040	4.225	<0.001^***^	0.091	0.249
Total indirect effect	0.090	0.028			0.038	0.150
**Model a**						
Indirect effect (X^a^ → depression →Y^b^)	0.077	0.026			0.027	0.130
Indirect effect (X → anxiety → Y)	0.011	0.009			−0.004	0.030
Indirect effect (X → depression → anxiety → Y)	0.003	0.002			−0.001	0.009
**Model b**						
Indirect effect (X → anxiety → Y)	0.014	0.011			−0.006	0.037
Indirect effect (X → depression → Y)	0.033	0.024			−0.012	0.080
Indirect effect (X → anxiety → depression → Y)	0.044	0.011			0.026	0.069

a X, Somatic symptom disorder.

b Y, Subjective cognitive decline.

c 95% Confidence Interval.

^***^ Significance at the 0.001 level (two-tailed).

CI, Confidence interval, LLCI, Lower limit of confidence interval, ULCI, Upper limit of confidence interval.

### 3.4 Moderated mediation model

**Figure 3:**
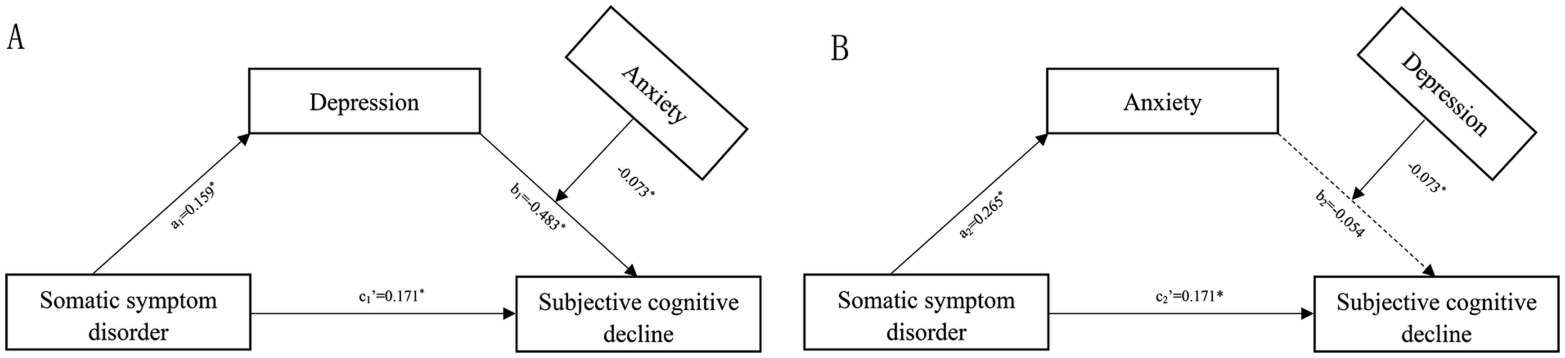
Moderated mediation model with anxiety as the moderator **(A)** or depression as the moderator **(B)**. Numbers associated with a, b, and c’ are unstandardized regression coefficients. *Significance at the 0.05 level (two-tailed).

In light of the results presented above, we further developed a moderated mediation model. With disease duration and Fazekas grade controlled, we found that anxiety moderated the mediating effect of depressive symptoms on the correlation between SSD and SCD (Figure 3A), whereas depressive symptoms did not modulate that of anxiety on the linkage between SSD and SCD (Figure 3B). The results revealed a significant overall positive correlation between depression and SCD (b1 = 0.483, 95% *CI*: 0.414 to 0.552), which was significantly modulated by anxiety symptoms (β = −0.073, 95% *CI*: −0.131 to −0.014) (Figure 3A), but no overall positive correlation between anxiety and SCD (b1 = 0.054, 95% *CI*: −0.017 to 0.125) (Figure 3B). Moreover, the effect of depression on SCD was greater in participants with severer anxiety symptoms.

## 4 Discussion

For the first time, this study examined the role of depression and anxiety in the correlation between SSD and SCD in an elderly Chinese population. Simple mediation analysis revealed that depression and anxiety separately mediated the SSD-SCD correlation. Serial mediation found a serial link between anxiety and depression in the association between SSD and SCD symptoms. Moderated mediation analysis showed that anxiety modulated the mediating role of depression in the SSD-SCD connection.

SSD refers to a spectrum of symptoms, such as pain, fatigue and gastrointestinal discomfort, which may occur alone or in combination (Wu et al., 2023). Patients with SSD may be afflicted with impairment in different cognitive domains. For instance, patients afflicted with pain often experience impaired executive function and attention, and the degree of impairment may be related to the severity of the pain (Phelps et al., 2021). Those afflicted with fatigue may suffer from impairments in processing speed, working memory, and learning (Michiels and Cluydts, 2001). Furthermore, SSD may exert multifactorial and complex effects on subjective cognitive function by affecting areas such as information processing speed, executive function, and attention. Some studies have explored the neurocognitive characteristics of SSD patients and found that SSD mainly impact their working memory, executive function, attention and visuospatial function (de Vroege et al., 2020; de Vroege et al., 2017). Consistently, our results suggest that patients with severer somatic symptoms may experience more pronounced subjective cognitive decline. These results suggest that clinicians should also evaluate subjective cognitive function in patients with SSD during their diagnosis and treatment, which may identify potential subjective cognitive decline in patients with SSD and prompt further interventions to avoid further deterioration of cognitive function.

In recent years, studies have documented local structural changes in some brain regions that may be associated with cognitive complaints in SSD patients (Kim et al., 2019). Studies of patients with SSD have reported an increase in mean gray matter volume in the bilateral posterior cerebellar lobes and found a significantly-increased functional connectivity of the gray matter with the postcentral gyrus, parietal lobe, cingulate gyrus and white matter (Liang et al., 2022). Other studies reveal that the structural changes in the cerebellum may affect the cognitive functions of SSD patients, such as attention and working memory (Phillips et al., 2015) and that local morphological changes in the frontal and anterior cingulate gyri of fibromyalgia patients may decrease their working memory (Luerding et al., 2008). Still others evidence that an increased functional connectivity between sensorimotor and dorsal attention networks is associated with impaired attention in patients with SSD (Kim et al., 2019). Overall, the structural changes in brain networks in patients with SSD are strongly associated with SCD. These findings may provide some insights into the relationship between SSD and SCD and guidance for the study, diagnosis and treatment of SSD and SCD.

Previous studies have documented a significant neurocognitive decline in SSD patients comorbid with depression (de Vroege et al., 2017). In the elderly population, the severity of depressive symptoms is closely related to the severity of SCD, not to objective cognitive decline (Zlatar et al., 2018), and depressive symptoms can significantly affect subjective cognitive functions such as executive function and attention and are associated with white matter hyperintensity lesions in the frontal cortex (Taylor et al., 2013). In this study, mental disorders such as depression and anxiety mediate the association between SSD and SCD, with depression playing a stronger mediating role in the association between SSD and SCD when compared with anxiety, which, consistent with previous studies, suggests that SSD patients with comorbid depression will experience worse subjective cognitive functional outcomes. Therefore, for SSD patients with comorbid mental disorders such as anxiety and depression, especially those with depressive symptoms, we should pay more attention to their possible cognitive decline and initiate comprehensive intervention for them.

It has been documented that anxiety has a curvilinear relationship with cognitive function in the elderly population, i.e., mild anxiety symptoms are associated with better cognitive ability, whereas severe anxiety symptoms are correlated with greater cognitive impairment and depressive symptoms are linearly correlated with cognition (Bierman et al., 2005). Previous studies have suggested that the relationship between SSD and SCD may be influenced by psychological factors but failed to perform any relevant mediation analysis. Some studies have explored the effects of comorbid depression and anxiety on neurocognitive function in patients with SSD and found that anxiety does not affect cognitive function in patients with SSD (de Vroege et al., 2017). Other studies have reported that patients with anxiety have some degree of subjective cognitive deficits (such as executive function, memory and attention). However, none of these studies have focused on the effect of anxiety on subjective cognitive function in patients with SSD (Castaneda et al., 2008; Tempesta and Serroni, 2013). In the current study, we evidenced that anxiety played a weak mediating role in SSD-SCD correlation. We performed a serial mediation analysis of anxiety and depression, which revealed an insignificant indirect effect path (SSD → depression → anxiety → SCD) but a significant alternative indirect effect path (SSD → anxiety → depression → SCD). This finding suggests that the deterioration of SSD symptoms may aggravate the anxiety symptoms, in turn worsening the depressive symptoms and SCD while SSD does not exert a direct effect on depression. Contrary to our hypothesis, this discrepancy may be related to the special regulatory effect of anxiety on cognitive function. However, further in-depth investigation is awaited to explore the impact of anxiety on cognitive function so as to provide further guidance for clinical diagnosis and treatment.

Interestingly, we found that the mediation analysis of the SSD-SCD correlation revealed that anxiety negatively moderated the depression-SCD pathway and that depression did not modulate the anxiety-SCD pathway. Though contrary to our hypothesis, the disparity may be accounted for by the following evidence. Previous literature has documented that patients with mild anxiety may have better cognition (Bierman et al., 2005) and that although the comorbidity of anxiety with depression may affect the cognitive function of patients, when analyzed separately, anxiety alone does not predict the level of cognitive function (Freedman et al., 2024). Therefore, it is of great importance for clinicians to incorporate into their decision-making process the severity of anxiety, its potential comorbidity with depression, and the impact of anxiety on SCD while diagnosing and treating SCD patients.

## 5 Limitations

Some limitations remain in this study. First, the study was cross-sectional and no causal relationship could be established between SSD, depression, anxiety and SCD. Second, the symptoms in this study were all assessed using a scale, and potential reporter bias cannot be ruled out. In addition, the sample size of this study was small and future investigations should recruit a larger sample size.

## 6 Conclusion

Despite the above limitations, this study provides important evidence for the pathway linking SSD to SCD in the Chinese elderly population. This finding will provide important information about the subjective perceptions of older adults and enable clinicians to understand SCD more deeply. It also suggests that researchers consider the effects of depression and anxiety on cognitive decline when investigating the etiological and psychological factors that may lead to cognitive decline. Future interventions targeting somatic symptoms, anxiety, and depression could help improve subjective cognitive decline.

## Data Availability

The raw data supporting the conclusions of this article will be made available by the authors, without undue reservation.
