# Supplementary material for: The impact of depression and anxiety on the correlation between somatic symptom disorder and subjective cognitive decline in the Chinese elderly population: an exploration by simple, serial, and moderated mediation models
Source: Front Psychol. 2025 Feb 14;16:1545325. doi: 10.3389/fpsyg.2025.1545325 (PMC11868093; doi:10.3389/fpsyg.2025.1545325)
Supplement: Supplementary file 1 [file Table_1.docx]

**Supplemental** **Table 1. The statistically significant variables in univariate analysis were further screened by multiple linear regression method**

| **Variables** | **B** | **β** | **t** | **P** | **VIF** | **95% CI** | |
| --- | --- | --- | --- | --- | --- | --- | --- |
|  |  |  |  |  |  | **Lower** | **Upper** |
| Age | 0.046 | 0.072 | 1.541 | 0.124 | 1.418 | -0.013 | 0.104 |
| Education level | -0.097 | -0.063 | -1.554 | 0.121 | 1.073 | -0.220 | 0.026 |
| Disease duration | 0.150 | 0.106 | 2.286 | **0.023^*^** | 1.403 | 0.021 | 0.280 |
| Fazekas grade | 0.854 | 0.177 | 4.137 | **＜0.001^***^** | 1.203 | 0.448 | 1.259 |
| Somatic symptom severity | 0.239 | 0.242 | 5.239 | **＜0.001^***^** | 1.400 | 0.149 | 0.328 |
| *R^2^* | 0.210 | | | | | | |
| *F* | 27.629 | | | | | | |
| D-W *value* | 2.268 | | | | | | |

*Significant at the 0.05 level (two-tailed); **Significant at the 0.001 level (two-tailed), VIF Variance inflation factor. 95% CI 95% Bias Corrected Confidence Interval.

**Supplemental Table 2. Analysis of the correlation between depression, anxiety, somatic symptom disorders and subjective cognitive decline.**

| **Item (*r* value)** | Depression | Anxiety | SSD *^a^* | SCD *^b^* |
| --- | --- | --- | --- | --- |
| Depression | 1.000 | 0.339^***^ | 0.169^***^ | 0.523^***^ |
| Anxiety | 0.339^***^ | 1.000 | 0.316^***^ | 0.287^***^ |
| SSD *^a^* | 0.169^***^ | 0.316^***^ | 1.000 | 0.374^***^ |
| SCD *^b^* | 0.523^***^ | 0.287^***^ | 0.374^***^ | 1.000 |

*a,* somatic symptom disorder, *b*, subjective cognitive decline;

***Significant at the 0.001 level (two-tailed).

| Path | Estimate | SE | t | *P* | 95%CI ^c^ | |
| --- | --- | --- | --- | --- | --- | --- |
|  |  |  |  |  | Lower | Upper |
| Total effect (c) | 0.260 | 0.045 | 5.742 | <0.001^***^ | 0.171 | 0.349 |
| X ^a^→ Depression →Y ^b^ |  |  |  |  |  |  |
| Direct effect (c_1_ʹ) | 0.181 | 0.038 | 4.709 | <0.001^***^ | 0.105 | 0.256 |
| Indirect effect (a_1_×b_1_) | 0.079 | 0.026 | n/a | n/a | 0.030 | 0.132 |
| Indirect effect (a_1_) | 0.159 | 0.052 | 3.036 | 0.003^**^ | 0.056 | 0.262 |
| Indirect effect (b_1_) | 0.500 | 0.033 | 15.278 | <0.001^***^ | 0.436 | 0.564 |
| X ^a^→ Anxiety →Y ^b^ |  |  |  |  |  |  |
| Direct effect (c_2_ʹ) | 0.202 | 0.046 | 4.440 | <0.001^***^ | 0.113 | 0.292 |
| Indirect effect (a_2_×b_2_) | 0.058 | 0.015 | n/a | n/a | 0.031 | 0.093 |
| Indirect effect (a_2_) | 0.265 | 0.050 | 5.297 | <0.001^***^ | 0.167 | 0.363 |
| Indirect effect (b_2_) | 0.219 | 0.041 | 5.323 | <0.001^***^ | 0.138 | 0.299 |

**Supplemental Table 3. Total, direct and indirect effect of somatic symptom disorder on subjective cognitive decline through depression and anxiety**

^a^ X, somatic symptom disorder; ^b^ Y, subjective cognitive decline; ^c^ 95% Bias Corrected Confidence Interval. *Significant at the 0.05 level (two-tailed); **Significant at the 0.01 level (two-tailed); ***Significant at the 0.001 level (two-tailed).
